# Antioxidants, Phytochemicals, and Cytotoxicity Studies on *Phaleria macrocarpa* (Scheff.) Boerl Seeds

**DOI:** 10.1155/2014/410184

**Published:** 2014-03-27

**Authors:** Ma Ma Lay, Saiful Anuar Karsani, Behrooz Banisalam, Sadegh Mohajer, Sri Nurestri Abd Malek

**Affiliations:** ^1^Institute of Biological Sciences, Faculty of Science, University of Malaya, 50603 Kuala Lumpur, Malaysia; ^2^University of Malaya Centre for Proteomics Research (UMCPR), University of Malaya, 50603 Kuala Lumpur, Malaysia

## Abstract

In recent years, the utilization of certain medicinal plants as therapeutic agents has drastically increased. *Phaleria macrocarpa* (Scheff.) Boerl is frequently used in traditional medicine. The present investigation was undertaken with the purpose of developing pharmacopoeial standards for this species. Nutritional values such as ash, fiber, protein, fat, and carbohydrate contents were investigated, and phytochemical screenings with different reagents showed the presence of flavonoids, glycosides, saponin glycosides, phenolic compounds, steroids, tannins, and terpenoids. Our results also revealed that the water fraction had the highest antioxidant activity compared to the methanol extract and other fractions. The methanol and the fractionated extracts (hexane, chloroform, ethyl acetate, and water) of *P. macrocarpa* seeds were also investigated for their cytotoxic effects on selected human cancer cells lines (MCF-7, HT-29, MDA-MB231, Ca Ski, and SKOV-3) and a normal human fibroblast lung cell line (MRC-5). Information from this study can be applied for future pharmacological and therapeutic evaluations of the species, and may assist in the standardization for quality, purity, and sample identification. To the best of our knowledge, this is the first report on the phytochemical screening and cytotoxic effect of the crude and fractionated extracts of *P. macrocarpa* seeds on selected cells lines.

## 1. Introduction

Herbal medicine plays a key role in the development of pharmaceuticals and thus there is a high demand in natural medicine for the global market. Although there are thousands of species listed as medicinal plants, only a small number are commercially used in traditional treatments. In this respect, there are very few in-depth scientific studies on the medicinal properties of plants. However, traditional herbal medicine is still prominent and is considered an important alternative to conventional medicine particularly in developing countries. Despite its well-known benefits,* Phaleria macrocarpa *(Scheff.) Boerl is still relatively unknown in terms of its biochemical constituents and biological activity.* P. macrocarpa* is a plant commonly used in East Asian herbal medicines.* P. macrocarpa *is used as a remedy for a variety of ailments such as cancer, diabetes mellitus, allergies, liver and heart diseases, kidney failure, blood diseases, high blood pressure, and stroke. It is also used to treat various skin diseases including acne [1, 2].

*P. macrocarpa* plantshave round, oval shaped seeds that have a diameter of approximately 1 cm. The seed is the most poisonous part of the plant, having higher toxicity levels than the stem, roots, and leaves.* P. macrocarpa *fruits and leaves are used in traditional medicine as a concoction. The seeds which have an unpleasant odor are usually used for the treatment of skin diseases. The compounds quercetin and naringin have been found in the seeds [3]. The essential oils of the seedsconsist of heptadecane, octadecane, diclosan, triclosan, vinyl laurate, and diocthyl ester [4]. Another study reported the presence of Mahkoside A and kaempferol 3-O-*β*-D-glucoside in the seeds [5]. Two novel compounds, 29-norcucurbitacin and desacetylfevicordin A, and three known 29-norcucurbitacin derivatives have also been isolated from the ethyl acetate fraction of* P. macrocarpa *seeds [6].

The ethanol extract of* P. macrocarpa* seeds exhibited toxicity towards T47D breast cancer cell lines (LC_50_15.12 ± 3.21 *μ*g/mL) through COX-2 inhibition [7]. Additionally, the ethanol extract of the seeds and the fruits' flesh have been shown to increase* p53 *gene expression but had no effect on Bcl-2 gene expression. Moreover, the n-hexane extract of the seeds had a greater effect in increasing* p53 *gene expression than that of the flesh of the fruit, but had no effect on Bcl-2 gene expression [8]. The ethanol extracts of* P. macrocarpa* seeds and fruits have been found to be nontoxic to human mononuclear peripheral normal cells but were slightly toxic to Vero cell lines [9].

Although* P. macrocarpa* has been used extensively in Indonesia, there is limited scientific research available on the biological properties of this plant in relation to its medicinal benefits. The present research investigated the total phenolic and flavonoid content and the antioxidative and cytotoxic activities of the crude and fractionated extracts of* P. macrocarpa* seeds.

## 2. Material and Methods

### 2.1. Plant Materials

Seeds of* Phaleria macrocarpa* (Scheff.) Boerl were collected from Yogyakarta, Indonesia, in December 2011. A voucher specimen (ID no. KLU 47923) was deposited into a repository at the Institute of Biological Sciences, Faculty of Science, University of Malaya, Malaysia. Samples were washed and dried in an oven at approximately 50°C. The seeds were then ground into powder and stored in airtight containers.

### 2.2. Preparation for Extraction

**Figure 1:**
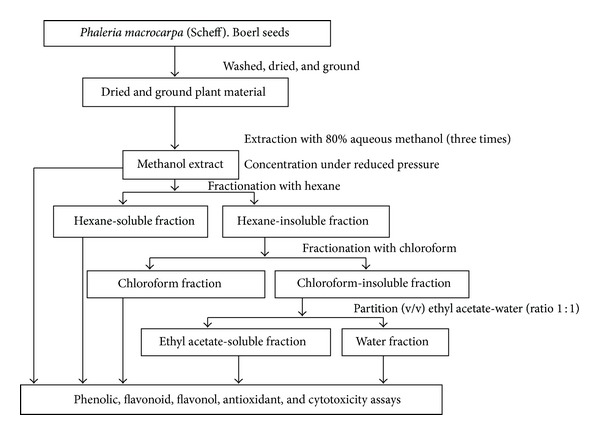
Extraction procedure and its fractionations of* P. macrocarpa* (Scheff.) Boerl seeds.

The dried powder was macerated with 80% aqueous methanol and extracted for 72 h before filtration (three times). The filtrate obtained was concentrated under reduced pressure (60 rpm at 37°C). This crude methanol extract was then fractionated, initially with hexane followed by chloroform. The chloroform insoluble fraction was subjected to partition with ethyl acetate and water (Figure 1). The methanol extract and its fractions (hexane, chloroform, ethyl acetate and water) were refiltered and evaporated at low pressure (60 rpm at 37°C) to remove excess solvent.

### 2.3. Phytochemical Screening Analysis

In order to classify the types of organic constituents present in the plant samples, preliminary phytochemical screening tests were carried out on the plant samples according to the qualitative and quantitative methods of Trease and Evans [10] and Sofowora [11]. The organic constituents that were investigated were those listed in Table 2. *α*-Amino acids, carbohydrates, cyanogenic glycosides, organic acids, reducing sugars, saponin glycosides, and starch content determination were carried out on water extracts. Alkaloids tests were used with 1% HCl extract. Determination of flavonoids, glycosides, phenolic compounds, and tannins were performed on the ethanol extract. Test for steroids was performed on the petroleum -ether extract.

### 2.4. Physiochemical Determination

The nutritional values of the* P. macrocarpa* seeds, including moisture, ash, fiber, protein, fat, carbohydrate contents, and energy value, were determined by using the methods of AOAC [12], Trease and Evans [10], and Harbone [13].

### 2.5. Determination of Moisture Content

The moisture content of the dried powder samples was determined by using the oven drying method [14, 15]. A clean dried crucible was weighed and 10 g of the dried* P. macrocarpa* seeds powder was placed in a beaker. The sample was dried in an electric oven at 105°C until all the moisture was removed from the sample and constant weight was achieved. The crucible containing the dried sample was weighed again and the loss of weight was recorded as the moisture content of the dried powdered* P. macrocarpa* seeds. The experiment was repeated three times. The moisture content (%) was calculated by using the following equation:

(1) $$\begin{array}{c} \text{Mositure}\;\text{percentag}\;\left(\%\right)=\frac{W_{2}-W_{1}}{W_{0}}\times 100, \end{array}$$

where *W*_1_ = weight of sample after drying (g), *W*_2_ = weight of sample before drying (g), and *W*_0_ = weight of the sample (g)

### 2.6. Determination of Ash Content

Ash content was determined by using the method of A.O.C.S [12]. Briefly, the ash value of the samples represented the inorganic residue when the organic matter has been burnt away. An accurately weighted amount (10 g) of the sample was placed in a preheated, cooled and weighed porcelain crucible. The crucible was heated carefully on a hot plate until the organic matter was dried and burnt off without flaming and finally heated in a furnace at 550 ± 50°C.

The percentage of ash content was calculated using the following formula:

(2) $$\begin{array}{c} \text{Percentage}\;\text{of}\;\text{Ash}\;\left(\%\right)=\frac{\text{Weight}\;\text{of}\;\text{Ash}}{\text{Weight}\;\text{of}\;\text{sample}}\times 100. \end{array}$$

### 2.7. Determination of Crude Fiber Content

The crude fiber content was determined by using the method of A.O.A.C [16]. Dried powdered sample was weighed (10 g) and extracted with petroleum ether (100 mL) three times. The extracted sample was air-dried and transferred to a round-bottomed flask. In the flask, 30 mL of sulphuric acid (0.1275 M) was added, followed by 170 mL of hot sulphuric acid. The solution was then refluxed for approximately 30 minutes and filtered through a Buchner funnel. The insoluble matter was washed with boiling water until the final filtrate was free from acid.

The residue was placed back into the flask with 30 mL of sodium hydroxide (0.313 M), and 170 mL of hot sodium hydroxide (0.313 M) was then added. The mixture was again refluxed for about 30 minutes and filtered using sintered glass. The residue was washed with 1% HCl and then washed again with boiling water until there was no acid present. The residue was finally washed with ethanol and ether and dried in an oven at 100°C and then weighed. This procedure was repeated until the fiber content was constant. The fiber content was calculated using the following equation:

(3) $$\begin{array}{c} \text{Percentage}\;\text{of}\;\text{Crude}\;\text{fiber}\;\left(\%\right)=\frac{\text{Loss}\;\text{in}\;\text{weight}}{\text{Weight}\;\text{of}\;\text{sample}}\times 100. \end{array}$$

### 2.8. Determination of Carbohydrate Content

Carbohydrate content was calculated by multiplying the reducing sugar content. The reducing sugar content was determined using the Fehling's reducing method of Lane and Eynon's [16]. Weighted sample (10 g) was placed into a 250 mL round-bottomed flask and 20 mL of sulphuric acid (0.5 M) was added. Reflux was then performed in a sand bath for 2.5 hours. The residue was washed after filtration with warm distilled water. The solution was then neutralized with sodium carbonate power and the mixture's volume was made up to 100 mL with distilled water. This was followed by titration with Fehling's solution, equal amounts of solution A (copper sulphate solution) and solution B (sodium potassium tartrate and sodium hydroxide solution) using methylene blue as indicator. The mixed Fehling's solution (5 mL) was pipetted into a conical flask and distilled water (5 mL) was added. The solution was then boiled for 15 seconds. Methylene blue indicator (a few drops) was then titrated with the solution until the colour changed from blue to green. The carbohydrate content was then calculated according to following equation:

(4) Percentage  of  carbohydrate  content(%)  =5×0.005×100×100×100V×10×W×0.9%,

where *V* = volume of sample solution (titration volume) and *W* = weight of powdered sample

### 2.9. Determination of Fat Content

Fat content of* P. macrocarpa* seeds was determined using the soxhlet extraction method [12]. A seed sample (10 g) was placed in a soxhlet extractor. Petroleum ether was then poured into the extractor and extraction was performed for 12 hours. The volume of the petroleum ether extract was reduced to 15 mL by evaporation. This was then dried at 105°C in an oven until constant weight was achieved. The fat content was then calculated by using the following equation:

(5) %  of  Fat  content  =Weight  of  fat  obtained  from  sample×100Weight  of  sample.

### 2.10. Determination of Protein Content

Protein content was determined using the A.O.A.C method [12]. Briefly, powdered samples (1 g), 50 mL of distilled water, 5 g of copper sulphate, and 15 mL of concentrated sulphuric acid were added to a Kjeldahl flask. The flask was partially closed by means of a funnel, and the content was digested by heating the flask at an inclined position in the digester. The mixture was heated for 30 minutes until there was 40 mL of clear 0.1 M standard sulphuric. A few drops of methyl red indicator were mixed into the clear solution. The flask was then placed below the condenser and the end of the adapter tube was dipped in the acid. The flasks were set up for Kjeldahl distillation and 70 mL of 40% sodium hydroxide was added through the funnel. The funnel was washed twice with 50 mL of distilled water. Distillation was then performed for one hour. The distilled ammonia was then nitrated with 0.1 M standard solution until the color changed from yellow to colorless. The experiment was repeated three times. The nitrogen content and protein content in the sample were calculated using the following relation:

(6) Percentage  of  Nitrogen  (%)  =(Vs−Vb)×MA×0.0140×100Weight  of  sample  (W)Protein  content=percentage  of  Nitrogen  ×6.25,

where *V*_*s*_ = volume in cm^3^ of standard acid used in the titration of sample, *V*_*b*_ = volume in cm^3^ of standard acid used in the blank titration, *M*_*A*_ = morality of standard acid solution in mol dm^−3^, and *W* = weight of the sample in grams.

### 2.11. Determination of Phenolic Content

The total phenolic content of the crude methanol and the fractionated extracts (hexane, chloroform, ethyl acetate, and water) was determined using the Folin-Ciocalteu method [17–20]. Briefly, 200 *μ*L of each extract solution of different concentrations was mixed with 1 mL of Folin-Ciocalteu reagent (1 : 10 diluted with H_2_O) and 800 *μ*L of Na_2_CO_3_ (75.05 g/L). The mixture was thoroughly shaken for 15 minutes and then held in a water bath at a temperature of 37°C. The solution was allowed to stand for 1 hour at room temperature in a dark place and the absorption was measured at 750 nm using a spectrophotometer. Distilled water was used as blank, and gallic acid (0–250 mg/L) was used to construct a standard calibration curve. Gallic acid concentration was established from the calibration curve, *y* = 0.0221*x* + 0.2189; *R* ^2^ = 0.9914.

### 2.12. Determination of Flavonoid Content

Total flavonoid content of* P. macrocarpa *seeds was measured using the methods of Ebrahimzadeh, Nabavi, and Ordonez [20–22] with minor modifications. To determine the flavonoid content, 1 mL of each sample was added to 0.1 mL of 10% Al (NO_3_)_3_ solution, 0.1 mL of 1 M potassium acetate, and 3.8 mL of methanol. The solution was thoroughly mixed using a vortex mixer for two to three minutes and then stood untouched for 10 minutes at room temperature. Absorbance was determined at 415 nm using a spectrophotometer. The total content of flavonoids was measured and expressed as quercetin equivalent on a dry weight basis (*y* = 0.0855*x* + 0.2004; *R* ^2^ = 0.9813).

### 2.13. Determination of Flavonol Content

The method of Kumaran [23] and Mbaebie [24] with slight modifications was used to measure the total flavonol content of the methanol extract. Briefly, 1 mL of extract was added to a centrifuge tube with 2 mL of prepared AlCl_3_ in ethanol and 3 mL of sodium acetate (50 g/L) solution. The mixture was stirred thoroughly with a vortex and was then incubated for 1 hour. The absorbance was measured with a spectrophotometer at 440 nm. A calibration curve was constructed using quercetin (1, 5, 10, 15, and 20 mg/mL). The total flavonol content was calculated using the calibration curve with the following equation,*y* = 0.0353*x* + 0.1456; *R* ^2^ = 0.9807.

### 2.14. Antioxidant DPPH Assay

Screenings of antioxidant activity of the crude methanol extract, chloroform, hexane, ethyl acetate, and water fractions of* P. macrocarpa* seeds were carried out by determination of DPPH free radical scavenging property using UV spectrophotometric methods [21, 25]. Based on this protocol, 50 *μ*L of test solutions from different dry extracts and concentrations (1, 5, 10, 15, and 20 mg/mL) was dissolved in water. This solution was then combined with 1.95 mL of DPPH methanol solution. After being mixed, solutions were kept at room temperature, in the dark for 30 minutes. After the reaction, the increase in absorbance was recorded at 517 nm. Methanol was used as a blank, DPPH solution was used as negative control (*A*_0_), and gallic acid was used as positive control. The antioxidant activity was expressed as an IC_50_ value. All experiments were carried out in triplicate. The scavenging effect was obtained from the following relation:

(7) $$\begin{array}{c} \text{Scavenging}\;\text{effect}\;\left(\%\right)=\frac{\left(A_{0}-A_{1}\right)\times 100}{A_{0}}, \end{array}$$

where *A*_0_ was the absorbance of the control reaction and *A*_1_ was the absorbance of the sample of the tested extracts. Gallic acid was used as standard. Percentage of inhibition was calculated using the following formula: % inhibition = [(*A*_negative_ − *A*_test_)/*A*_negative_] × 100 (*A* is absorbance).

### 2.15. Cytotoxicity Screening

#### 2.15.1. Cell Culture and Culture Medium

Human cervical carcinoma cells (Ca Ski), hormone-dependent breast carcinoma cells (MCF-7), human breast adenocarcinoma cells (MDA-MB231), human ovarian carcinoma cells (SKOV-3), human colon carcinoma cells (HT-29), and noncancer human fibroblast cells (MRC-5) were purchased from the American Tissue Culture Collection (ATCC, USA).

HT 29, Ca Ski, and MCF-7 cells were maintained in RPMI 1640 medium (Sigma), MDA-MB231 and SKOV-3 cells in Dulbecco's Modified Eagle's medium (DMEM, Sigma), and MRC-5 cells in Eagle's Minimum Essential medium (EMEM, Sigma), supplemented with 10% fetal bovine serum (FBS, PAA Lab, Austria), 100 *μ*g/mL penicillin or streptomycin (PAA Lab, Austria), and 50 *μ*g/mL kanamycin/amphotericin B (PAA Lab, Austria). The cells were cultured in a CO_2_ incubator (5%) and kept at 37°C in a humidified atmosphere. The cultures were subcultured every 2-3 days and checked frequently under an inverted microscope (Leica, Germany) for any contamination.

#### 2.15.2. MTT Cell Proliferation Assay

MTT [3-(4, 5-dimethylthiazol-2-yl)-2, 5-diphenyl tetrazolium bromide)] assay was performed on the cultured cells in 96-well plates according to the method of Mosmann [26, 27]. Briefly, cells were cultured to confluence. They were then centrifuged at 1,000 rpm for 5 minutes and resuspended with 1.0 mL of growth medium. The density of the viable cells was counted using 0.4% trypan blue exclusion dye in a haemocytometer with a microscope. The cells were then seeded into microtiter plates and incubated in a CO_2_ incubator at 37°C for 24 hrs. After reaching 70–80% confluence, each extract at concentrations of 1, 10, 25, 50, and 100 *μ*g/mL (in 200 *μ*L of 10% media) was added to the respective wells containing the cells. Wells with untreated cells were used as the negative control and cells exposed to doxorubicin were used as positive control. After 24, 48, and 72 hours, 10 *μ*L MTT stock solution was added to each well. OD was then determined by measuring absorbance at 540 nm using an ELISA microplate reader. The percentage of inhibition (%) was calculated according to the following formula:

(8) Percentage  of  inhibition  (%)  =OD  control−OD  sample×100%OD  control.

Cytotoxicity of each sample is expressed as ±IC_50_ value. The extract that gave IC_50_ of 30 *μ*g/mL or less was considered active [26].

### 2.16. Gas Chromatography-Mass Spectrometry (GC-MS)

GC-MS was used to determine the molecular weight of the components collected and the purity of the collected extracts. Running conditions were as follows: oven temperature was programmed with an initial temperature of 100°C and increased at a ramp rate of 5°C/min and reached a final temperature of 300°C. The carrier gas or mobile phase used was helium and a flow rate of 1 mL/min was programmed. The mass spectrometry mode used was electron ionization (EI) mode with a current of 70 eV. The injection mode was programmed with a sample injection volume of 1 *μ*L with a split mode at a ratio of 1 : 20. The injection port temperature was set to 230°C and the detector/interface temperature was set to 250°C. The results were collected for 40 minutes. The total ion chromatogram obtained was autointegrated using ChemStation and the chemical compounds or components were analysed by comparison with the supplied mass spectral database (NIST 05 Mass Spectral Library, USA).

### 2.17. Statistical Analysis

All data for each test are the average of triplicate experiments for comparison of values and were recorded as the mean ± standard deviation using Microsoft Excel software and statistical data analyses were performed using SPSS software.

## 3. Results

### 3.1. Extraction

The extracts were concentrated using a rotary evaporator (Buchi, USA) under reduced pressure at 35°C. The yield of extracts from* P. macrocarpa* seeds is shown in Table 1. The highest yield from* P. macrocarpa *seeds was the hexane fraction (9.47%).

### 3.2. Preliminary Phytochemical Studies

In order to determine the types of phytoorganic constituents present in* P. macrocarpa* seeds, a preliminary phytochemical investigation was carried out according to conventional methods. The results obtained from these experiments are summarized in Table 2.

The phytochemical tests showed that there were secondary metabolites, including carbohydrate, flavonoids, glycosides, saponin glycosides, phenolic compounds, steroids, tannins, and terpenoids, present in different extracts of* P. macrocarpa* seeds. Small amounts of alkaloids, *α*-amino acids, cyanogenic glycosides, organic acids, reducing sugars, and starches were also found to be present.

The main constituents such as flavonoids, glycosides, saponin glycosides, phenolic compounds, steroids, tannins, and terpenoids present in* P. macrocarpa* seeds may contribute to the presence of bioactivities such as antibacterial, an analgesic, an antifungal, an anti-inflammatory agent, and cytotoxicity. Moreover, the toxic chemical constituents, cyanogenic glycosides, were also present in the seeds.

### 3.3. Physicochemical Studies

**Figure 2:**
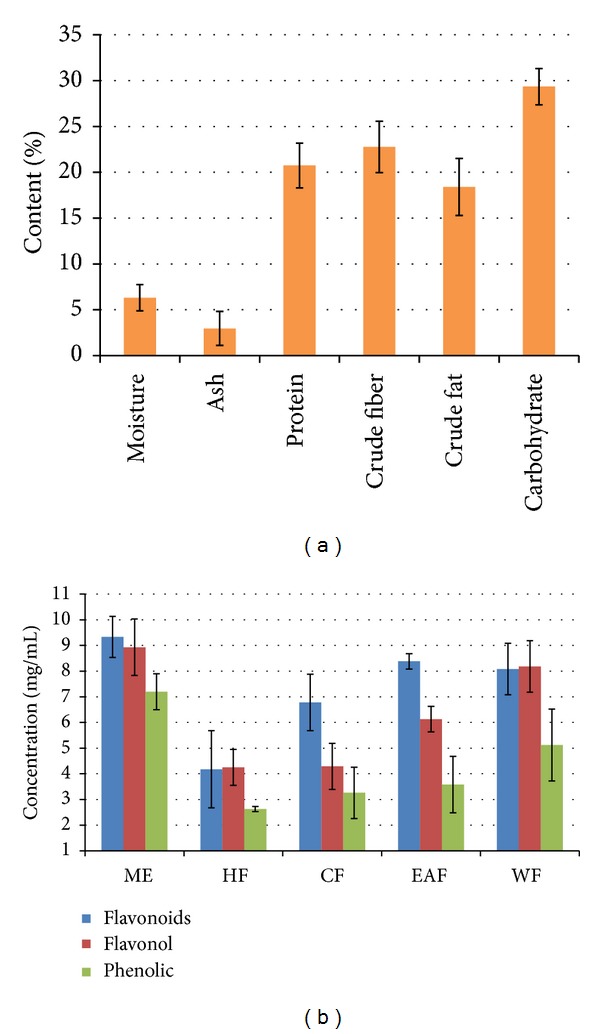
(a) The percentage of moisture, ash, protein, crude fiber, crude fat, and carbohydrate contents on* P. macrocarpa* Boerl seeds (b) Total amount of concentration of flavonoid, flavonol, and phenolic contents of* P. macrocarpa *seeds. ME: methanol extract, HF: hexane fraction, EAF: ethyl acetate fraction, CF: chloroform fraction,WF: water fraction.

The determination of nutritional values such as moisture, ash, fiber, protein, fat, and carbohydrate contents was carried out using the A.O.A.C method as well as the Lane and Eynon titration method and the results obtained are shown in Figure 2(a) and Table 3. Different physiochemical parameters for the purpose of standardization such as moisture (6.31 ± 1.43%), ash (2.96 ± 1.86%), protein (20.73 ± 2.44%), crude fiber (22.76 ± 2.79%), crude fat (18.4 ± 3.11%), and carbohydrate (29.34 ± 1.98%) were determined.

### 3.4. Flavonoid, Flavonol, and Phenolic Determinations

It was found that the methanol extract had the highest flavonoid content (9.33 ± 0.8 mg/mL), followed by ethyl acetate, water, chloroform, and hexane fractions, which were 8.38 ± 1.0, 8.08 ± 0.3, 6.78 ± 1.1, and 4.18 ± 1.5 mg/mL, respectively.

The methanol extract (8.93 ± 1.1 mg/mL) of* P. macrocarpa* seeds exhibited the highest amount of total flavonol content, followed by water, ethyl acetate, chloroform, and hexane fractions, which were 8.93 ± 1.0, 6.13 ± 0.5, 4.29 ± 0.9, and 4.29 ± 0.7 mg/mL, respectively.

The total phenolic content of the* P. macrocarpa *seeds extracts and fractions was expressed as gallic acid equivalents. The methanol extract of* P. macrocarpa *seeds exhibited the highest amount (7.20 ± 0.7 mg/mL) of total phenolics, followed by water, ethyl acetate, chloroform, and hexane fractions, which were 5.12 ± 1.4, 3.58 ± 1.1, 3.26 ± 1.0, and 2.63 ± 0.1 mg/mL, respectively.

These data are shown in Figure 2(b) and Table 4.

### 3.5. DPPH (2, 2-Diphenyl-1-picryl-hydazyl) Antioxidant Assay

**Figure 3:**
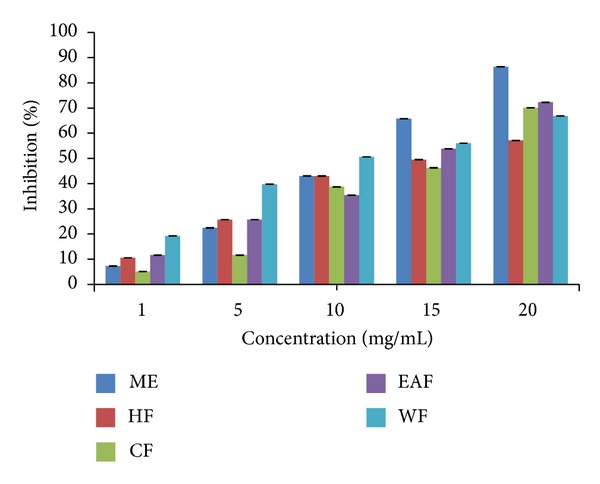
Percentage of inhibition of DPPH free radical scavenging of* P. macrocarpa* seeds. ME: methanol extract, HF: hexane fraction, EAF: ethyl acetate fraction, CF: chloroform fraction,WF: water fraction.

DPPH assay was used to determine the free radical scavenging ability of extracts and fractions of* P. macrocarpa *seeds and to determine the antioxidant activity of its phytoconstituents. It is important to note that a lower IC_50_ value equals a higher scavenging activity. The scavenging activity was presented as the percentage of inhibition of DPPH free radicals (Figure 3 and Table 4). Based on IC_50_ values, the samples can be ranked in the following descending order: water fraction > methanol extract > ethyl acetate fraction > hexane fraction > chloroform fraction. The results revealed that the water fraction was most active for antioxidant activity compared to other fractions. The radical scavenging effect was found to increase with increasing concentrations.

### 3.6. Cytotoxicity Screening of MTT Cell Proliferation Assay

The methanol and fractionated extracts (hexane, chloroform, ethyl acetate, and water) were investigated for cytotoxic effects in human cervical adenocarcinoma cells (Ca Ski), human hormone-dependent breast carcinoma (MCF-7), human colon adenocarcinoma cells (HT-29), human ovarian carcinoma cells (SKOV-3), human hormone-dependent breast carcinoma (MDA-MB231), and normal cells (MRC-5) using MTT cells proliferation assay at 24 hrs, 48 hrs, and 72 hrs, respectively.

The methanol extract of* P. macrocarpa* seeds showed excellent cytotoxic effects with IC_50_ value of 8.2 ± 4.66 *μ*g/mL in Ca Ski cells at 24 hrs and 12.0 ± 2.2, 8.5 ± 1.68, and 3.0 ± 2.50 *μ*g/mL in MCF-7 cells at 24 hrs, 48 hrs, and 72 hrs, respectively. The methanol extract also showed good cytotoxic activity in HT-29 cells with IC_50_ values of 29.3 ± 2.26, 25.0 ± 1.35, and 21.5 ± 3.30 *μ*g/mL at 24 hrs, 48 hrs, and 72 hrs, respectively, in Ca Ski cells with IC_50_ values of 19.7 ± 0.92 *μ*g/mL, and in SKOV-3 cells with IC_50_ values of 16.5 ± 2.52, 22.1 ± 2.47, and 36.0 ± 3.55 *μ*g/mL at 24 hrs, 48 hrs, and 72 hrs, respectively. However, this extract had no cytotoxic effect in MDA-MB231 cells with IC_50_ >100 *μ*g/mL, and the extract also had low cytotoxic effect in the MRC-5 cells with IC_50_ > 50 *μ*g/mL.

The hexane fraction of* P. macrocarpa* seeds showed moderate cytotoxic effects with IC_50_ values of 45.2 ± 1.49 and 55.5 ± 1.97 *μ*g/mL at 24 hrs and 48 hrs on MCF-7, 40.0 ± 3.15 *μ*g/mL in HT-29 cells at 24 hrs, and 40.5 ± 3.52 and 50.0 ± 3.02 *μ*g/mL in SKOV-3 at 24 hrs and 48 hrs. In addition, the hexane fraction also displayed low cytotoxic effect in MCF-7 at 72 hrs, HT-29 at 48 hrs and 72 hrs, and in SKOV-3 at 72 hrs. The IC_50_ value of treatment with hexane fraction in MCF-7 was 72.5 ± 1.52 *μ*g/mL for 72 hrs exposure. IC_50_ values in HT-29 were 64.0 ± 2.03 and 75.0 ± 3.14 *μ*g/mL at 48 hrs and 72 hrs, while IC_50_ value in SKOV-3 was 70.3 ± 3.53 *μ*g/mL at 72 hrs. In contrast, the hexane fraction exhibited no cytotoxic effects in MDA-MB231 cells or MRC-5 cells.

The chloroform fraction of* P. macrocarpa* seeds exhibited the highest cytotoxic effect with IC_50_ value of 10.0 ± 1.31, 8.2 ± 1.04, and 22.0 ± 1.86 *μ*g/mL at 24 hrs, 48 hrs, and 72 hrs in Ca Ski cells, 9.5 ± 2.95, 8.7 ± 1.59, and 21.0 ± 1.98 *μ*g/mL at 24 hrs, 48 hrs, and 72 hrs on HT29, and 24.8 ± 2.06, 16.5 ± 3.21, and 9.00 ± 2.6 *μ*g/mL in SKOV-3 cells. The chloroform fraction exhibited moderate cytotoxic effect in MCF-7 with IC_50_ value of 57.5 ± 2.64, 40.0 ± 1.48, and 46.5 ± 3.45 *μ*g/mL for 24 hrs, 48 hrs, and 72 hrs, respectively. In contrast, the chloroform fraction had no cytotoxic effect against the MDA-MB231 cells and normal MRC-5 cells with IC_50_>100 *μ*g/mL.

**Figure 4:**
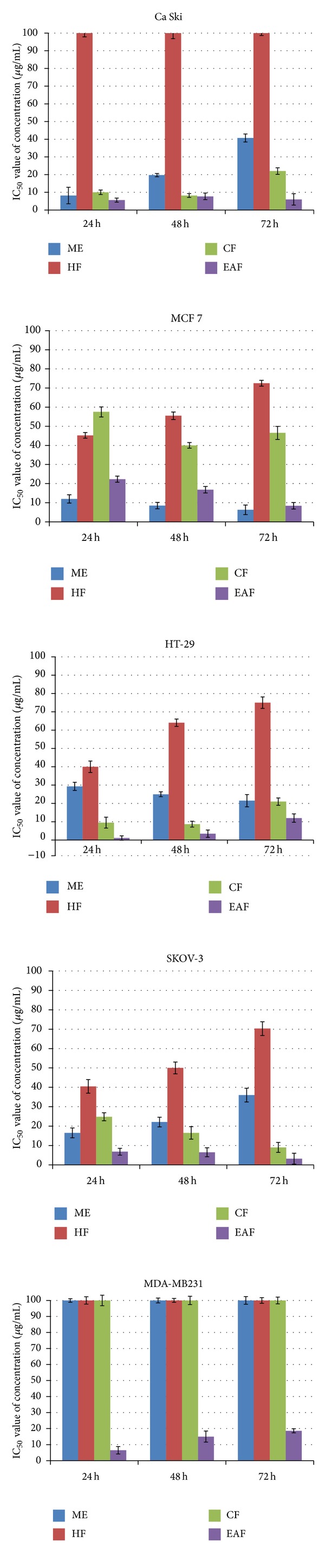
*In vitro*, cytotoxic effects of* P. macrocarpa *seedson Ca Ski, MCF-7, HT-29, SKOV-3, and MDA-MB231 cell lines. ME: methanol extract, HF: hexane fraction, EAF: ethyl acetate fraction, CF: chloroform fraction,WF: water fraction. Each value is expressed as mean ± standard deviation of three measurements.

The ethyl acetate fraction of* P. macrocarpa* seeds exhibited the highest cytotoxic effect with IC_50_ < 25 *μ*g/mL in SKOV-3 cells, MDA-MB 231 cells, MCF-7 cells, and Ca Ski cells. On the other hand, the ethyl acetate fraction exhibited low cytotoxic effect in MRC-5 normal cells with IC_50_ value of 35 *μ*g/mL. The ethyl acetate fraction exhibited the highest cytotoxic effect in all selected cells (Ca Ski, MCF-7, HT-29, and MDA-MB231). The water extract showed no cytotoxic effect against all selected cancer cell lines with IC_50_>100 *μ*g/mL and exhibited a mild cytotoxic effects against MRC-5 cells. The corresponding data is shown in Figure 4 and Table 5.

### 3.7. Characterization and Identification of Hexane and Chloroform Fractions

**Figure 5:**
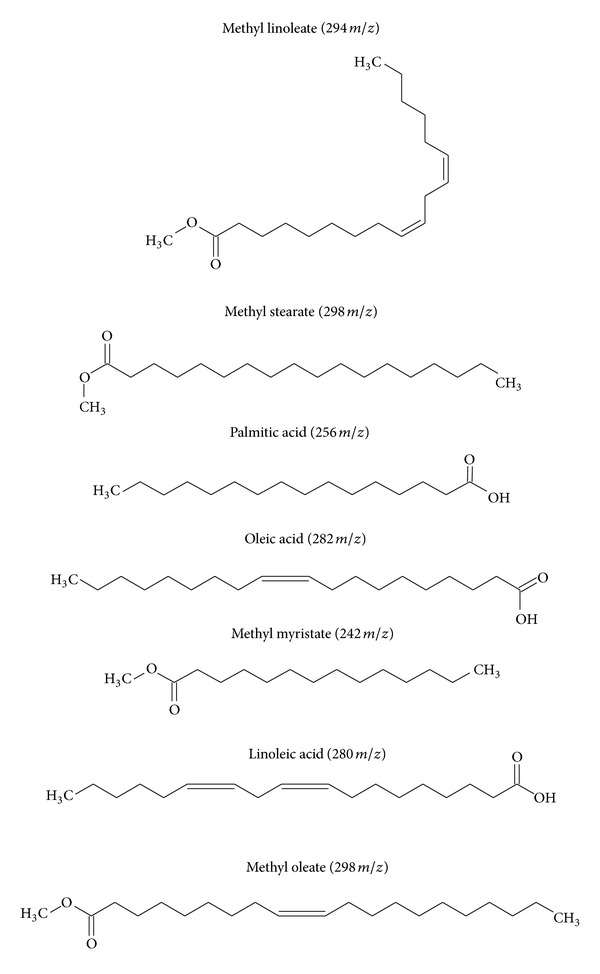


Six compounds were identified from the hexane fraction of* P. macrocarpa *seeds using GC-MS. They were methyl stearate, oleic acid, methyl oleate, linoleic acid, methyl linolenate, and palmitic acid. Moreover, a GC/MS analysis of the chloroform fraction of* P. macrocarpa *seeds showed the presence of methyl myristate, palmitic acid, methyl oleate, methyl linoleate, oleic acid, and (*z*)- and 9,17-octadecadienal, (*z*). A comparison with the NIST mass spectral library (NIST 05 MS library, 2002) and Adams (2001) confirmed the identity of the compounds. The chemical structures of the identified compounds in the hexane and chloroform fractions of* P. macrocarpa *seeds are as shown in Figure 5.

## 4. Discussion

A longer shelf life can be achieved by reducing moisture content. Thus, moisture content is a critical factor for the stability of an extract. Moisture enhances fungal and bacterial growth, therefore decreasing the longevity of the extract. The time it takes for plant material to deteriorate depends on how much water the plant material contains.

Carbohydrates play a vital role in the immune system, fertilization, pathogenesis, blood clotting, and human development. Foods that contain carbohydrates can raise blood glucose and the three main types of carbohydrate are starches, sugars, and fiber. A macronutrient protein consists of amino acids which is required for proper growth and human body function. The seeds were found to contain a high level of crude fiber which is a potential source of phenolic antioxidants. The major components of fibers are cellulose, hemicelluloses, lignin, *β*-glucans, gums, and pectin and hydrocolloids. These components can behave as proantioxidants [28, 29]. In our present physicochemical study,* P. macrocarpa* seeds were mainly made up of carbohydrates, followed by crude fiber, proteins, fat, moisture, and ash. High-protein and low-carbohydrate diets are often effective for weight loss. Dietary fiber can aid in digestion, is helpful for weight management, and reduces constipation. However, many high-protein and low-carbohydrate foods are low in fiber. Women aged 50 and older need at least 21 grams of fiber daily, whilst those aged 19 to 50 require 25 grams, men aged 50 and older need 30 grams, and those between the age of 19 to 50 should consume at least 38 grams of fiber.

Steroids possess biological activities which are insecticidal, cardiotonic, and antimicrobial activities and these are potentially useful for development into therapeutic drugs. Tannins are known to be important for their antiviral, antibacterial, antiparasitic effects, anti-inflammatory, antiulcer. and antioxidant properties [30–33]. Saponins have been found to have antimicrobial, anti-inflammatory, antifeedant, and hemolytic effects [34, 35]. Some alkaloids have been reported to have anticancer and antiviral activity and saponins have been reported to be cardiotonic, while flavonoids have anticancer and anti-inflammatory activity [10, 36]. The presence of tannins may be responsible for the ability of* P. macrocarpa *seeds to be used in the treatment of diseases such as diabetes, diarrhea, and dysentery. Our preliminary phytochemical studies revealed that the chemical components of* P. macrocarpa *seeds also include flavonoids, phenolics, steroids, tannins, terpenoids, glucosides, saponins, and carbohydrates.

Previous researchers reported that kaempferol, myricetin, naringin, quercetin, rutin [3], 29-norcucurbitacin derivatives, fevicordin A, fevicordin A glucoside, fevicordin D glucoside [6], mahkoside A, dodecanoic acid, palmitic acid, des-acetyl flavicordin-A, flavicordin-A, flavicordin-D flavicordin-A glucoside, ethyl stearate, lignans and sucrose [37, 38], mangiferin (a C-glucosylxantone), kaempferol-3-o-*β*-D-glucoside, dodecanoic acid, palmitic acid, ethyl stearate, sucrose [5], pinoresinol, lariciresinol, matairesinol, alkaloids and saponins [37, 39], saponins, alkaloids, polyphenolics, phenols, flavanoids, lignans, tannins [40–42], icariside C3, mangiferin, gallic acid, phalerin, glycoside (3,4,5, trihydroxy-4-methoxy-benzophenone-3-O-*β*-D-glucoside) [43], and 2,4′,6, trihydroxy-4-methoxy-benzophenone-3-O-*β*-D-glucoside [44] were isolated from different parts of* P. macrocarpa.* The hexane extract of the seeds was found to contain methyl myristate, methyl stearate, oleic acid, methyl oleate, linoleic acid, methyl linoleate, palmitic acid, methyl palmitate, 6-octadecenoic acid, and 9,17-octadecadial, *β*-sitosterol as major components. A further 30% to 40% of components were not identified as these were polar compounds that require HPLC analysis. Other compounds reported in the literature for the seeds, leaves, and fruits were not identified in this investigation. It is probable that the components reported in the literature may be present in other fractions (methyl acetate, CHCl_3,_ and water fractions).

Phenolics are able to transfer protons to radicals [45]. DPPH, which provides free radicals, is normally a blue-violet color. However, the DPPH turned yellow after it was converted to 1, 1-diphenyl-2-picrylhydrazine, which has less free-radical activity. Hendra et al. reported that the IC_50_ values of free radical and reducing power in methanol with HCl of* P. macrocarpa* seeds were 245.0 ± 1.94 and 150.2 ± 1.28 [42]. In our investigation using DPPH assay, the water fraction displayed the highest IC_50_ value followed by methanol, ethyl acetate, hexane, and chloroform fraction.

The presence of flavonoids in* P. macrocarpa *seeds may be responsible for the traditional use of the plants in treating cancer, inflammations, and allergies. Recently, it has been shown that phenolic compounds in crude methanol and ethyl acetate extract of* P. macrocarpa* leaves displayed good antioxidant and antimicrobial activites [46]. Hendra et al. (2011) examined the amount of total phenolic (47.7 ± 1.04 mg gallic acid equivalent/g DW) and the amount of flavonoid (35.9 ± 2.47 mg rutin equivalent/g DW) contents in the methanol extract with HCl of* P. macrocarpa* seeds [42]. In this study, the total flavonoids content was higher than that in the results previously reported by Rohyami [47] and phenol, flavonol, and flavonoid contents were the highest in the aqueous methanol extract followed by ethyl acetate, water, chloroform, and hexane extracts

MTT assay is a sensitive and reliable colorimetric assay that uses quantitative measurements to calculate the viability, proliferation, and activation of cells. This method is commonly applied to screen anticancer agents [48]. The most well-known methods used to calculate cytotoxicity are the neutral red (NR) uptake and dimethylthiazole-diphenyl tetrazolium bromide (MTT) metabolism [27, 49]. Earlier investigations showed that the ethanol extract of* P. macrocarpa* seeds and fruit meat were not toxic to normal human cells, but slightly toxic to a Vero cell line [9]. The ethanol extract of* P. macrocarpa* seed fruit also showed toxicity towards T47D breast cancer cell line through COX-2 expression inhibition [7]. Desacetylfevicordin A has been isolated from the ethyl acetate extract of* P. macrocarpa* seeds and this compound displayed excellent cytotoxicity in brine shrimp [43]. The ethyl acetate of* P. macrocarpa* seeds exhibited mild cytotoxic effect against HepG2 cells (IC_50_ values between 30 and 60 *μ*g/mL) [46]. Previous studies showed that the cytotoxic activity of the seeds on HT-29, MCF-7, Hela, and Chang liver cells lines were 38.4 ± 0.37, 25.5 ± 1.37  29.5 ± 1.0 and 67.8 ± 0.27, receptively [42]. In our study, the methanolic extract of* P. macrocarpa* seeds was found to pose cytotoxic effect against HT-29, MCF-7, Cas Ki, and SKOV-3 cell lines (IC_50_ values giving from 1.1 ± 1.20 to 36.0 ± 3.55) and mild toxicity on normal cell lines. There were significant cytotoxic effects on selected cancer cells lines that were both time- and dose-dependent manner due to the presence of many secondary metabolites.

The phytochemical screening also indicated the presence of a small amount of cyanogenic glycosides. Cyanogenic glucosides which can cause acute cyanide poisoning cause rapid respiration and pulse, decrease the blood pressure, and induce vomiting, diarrhea, headache, dizziness, and so on. Thus, these components have to be removed (usually by boiling) prior to consumption for therapeutic purposes.

## 5. Conclusion

*P. macrocarpa *seeds exhibited antioxidant and cytotoxic activities. It is highly probable that these activities are due to the presence of phenolic and flavonoid compounds in appreciable amounts in the plant. Furthermore, the cytotoxicity activity suggested that the seed may contain a potential anticancer agent. The outcome of this study is encouraging, demonstrating the potential for the* P. macrocarpa *as a source of multiple therapeutic agents.

## Figures and Tables

**Table 1:** Yield of methanol extract and its fractions from *P. macrocarpa *of seeds.

Extract and fractions	Yield (g)	Percentage of yield (%)
Methanolic extract (ME)	198.7	11.99
Hexane fraction (HF)	158.7	9.47
Chloroform fraction (CF)	7.2	0.43
Ethyl acetate fraction (EAF)	6.2	0.37
Water fraction (WF)	Freeze dry	

**Table 2:** Results of chemical constituents of *P. macrocarpa* seeds.

No.	Tests	Reagents	Observation
1	Alkaloids	Wagner's reagent Mayer's reagent Dragendorff's reagent Sodium picrate solution	+ + + +
2	*α*-Amino acids	Ninhydrin reagent	+
3	Carbohydrates	10% *α*-naphthol and conc: H_2_SO_4_	++
4	Cyanogenic glycosides	Sodium picrate solution	+
5	Flavonoids	Mg and conc: HCl	++
6	Glycosides	10% lead acetate	++
7	Organic acids	Bromothymol blue	+
8	Phenolic compounds	1% FeCl_3_	++
9	Reducing sugars	Fehling's solutions A and B	+
10	Saponin glycosides	Distilled water	++
11	Starch	Iodine solution	+
12	Steroids	Acetic anhydride and conc: H_2_SO_4_	++
13	Tannins	1% Gelatin	++
14	Terpenoids	Acetic anhydride and conc: H_2_SO_4_	++

+++: large amount; ++: medium amount; +: small amount; −: absent; +: present.

**Table 3:** The percentage of moisture, ash, protein, crude fiber, crude fat, and carbohydrate contents and the amount of nutrition values on *P. macrocarpa* seeds.

Tests parameter	Percentage of contents (%)
Moisture	6.31 ± 1.43
Ash	2.96 ± 1.86
Protein	20.73 ± 2.44
Crude fiber	22.76 ± 2.79
Crude fat	18.40 ± 3.11
Carbohydrate	29.34 ± 1.98

**Table 4:** Total flavonoid, total flavonol, total phenolic contents, and result of DPPH free radical scavenging property of *P. macrocarpa* seeds.

Tests	ME (mg/mL)	HF (mg/mL)	CF (mg/mL)	EAF (mg/mL)	WF (mg/mL)
Flavonoid	9.33 ± 0.8	4.18 ± 1.5	6.78 ± 1.1	8.38 ± 0.3	8.08 ± 1.0
Flavonol	8.93 ± 1.1	4.25 ± 0.7	4.29 ± 0.9	6.13 ± 0.5	8.18 ± 1.0
Phenolic	7.20 ± 0.7	2.63 ± 0.1	3.26 ± 1	3.58 ± 1.1	5.12 ± 1.4
DPPH	11.50 ± 0.03	15.00 ± 0.04	15.75 ± 0.04	14.00 ± 0.05	9.75 ± 0.03

ME: methanol extract; HF: hexane fraction; EAF: ethyl acetate fraction; CF: chloroform fraction; WF: water fraction.

**Table 5:** *In vitro*, cytotoxic effects of methanol extract and its fractions of *P. macrocarpa *cancer cells lines and normal human fibroblast breast cells line.

Cell line	Incubation periods (hours)	ME	HF	CF	EAF	Doxorubicin
	24	8.2 ± 4.66	≥100	10.0 ± 1.31	5.6 ± 1.17	0.92 ± 0.64
Ca Ski	48	19.7 ± 0.92	≥100	8.2 ± 1.04	7.7 ± 1.85	0.69 ± 1.05
	72	40.7 ± 2.26	≥100	22.0 ± 1.86	6.0 ± 3.22	0.45 ± 0.99

	24	12.0 ± 2.2	45.2 ± 1.49	57.5 ± 2.64	22.3 ± 1.58	1.05 ± 1.08
MCF7	48	8.5 ± 1.68	55.5 ± 1.97	40.0 ± 1.48	16.8 ± 1.70	0.12 ± 0.69
	72	6.3 ± 2.50	72.5 ± 1.52	46.5 ± 3.45	8.4 ± 1.71	0.92 ± 1.70

	24	29.3 ± 2.26	40.0 ± 3.15	9.5 ± 2.95	1.1 ± 1.20	0.92 ± 0.72
HT29	48	25.0 ± 1.35	64.0 ± 2.03	8.7 ± 1.59	3.5 ± 2.00	0.32 ± 2.16
	72	21.5 ± 3.30	75.0 ± 3.14	21.0 ± 1.98	12 ± 2.28	0.88 ± 0.94

	24	16.5 ± 2.52	40.5 ± 3.52	24.8 ± 2.06	6.8 ± 1.8	1.02 ± 1.79
SKOV-3	48	22.1 ± 2.47	50.0 ± 3.02	16.5 ± 3.21	6.5 ± 2.3	0.32 ± 0.97
	72	36.0 ± 3.55	70.3 ± 3.53	9.0 ± 2.6	3.2 ± 2.81	0.62 ± 0.98

	24	≥100	≥100	≥100	6.5 ± 2.3	0.43 ± 0.50
MDA-MB231	48	≥100	≥100	≥100	15 ± 3.43	0.59 ± 1.01
	72	≥100	≥100	≥100	18.6 ± 1.26	0.95 ± 1.32

ME: methanol extract; HF: hexane fraction; EAF: ethyl acetate fraction; CF: chloroform fraction; WF: water fraction. Doxorubicin was used as positive control.

Each value is expressed as mean ± standard deviation of three measurements.

## Abbreviations

EAF: Ethyl acetate fraction
ME: Methanol extract
HF: Hexane fraction
CF: Chloroform fraction
WF: Water fraction
g: Gram
mg: Milligram
mL: Milliliter.
